# Validation of the arabic version of the EuroQol-5-dimension 5-level (EQ-5D-5 L) in patients with spinal degenerative diseases

**DOI:** 10.1186/s12889-024-18367-3

**Published:** 2024-04-23

**Authors:** Ahmed Shawky Abdelgawaad, Mohammad El-Sharkawi, Ahmed Mahmoud Sarhan, Mohammed Abdelghafour Hassanien, Mirette Aziz

**Affiliations:** 1 Department of Orthopaedic and Trauma Surgery, Assiut University Hospitals, Assiut, Egypt; 2 Department of Public Health & Community Medicine, Assiut University, Assiut, Egypt

**Keywords:** Validity, EQ-5D-5L, Quality of life, Arabic

## Abstract

**Objective:**

This study aims to test the reliability and validity of the translated Arabic version of EQ-5D-5 L.

**Methods:**

The study was conducted on 100 patients operated upon for degenerative spine diseases coming for follow up in the outpatient clinic of a Tertiary care hospital. Test-retest reliability was assessed by completing the self-administered tool in two follow up visits, one week apart, by 50 patients. Internal consistency was evaluated by Cronbach’s alpha. Intra-class correlation coefficients and kappa statistics were performed to test for the agreement between the two ratings. Criterion validity was assessed by comparing the responses of 100 patients to the EQ-5D-5 L with scores of two validated questionnaires; the Arabic version of the Oswestry disability index and the Arabic version of short-form health survey-36. The construct validity was assessed using known-groups comparison to test for hypothesized differences concerning demographic and clinical variables.

**Results:**

The Arabic version of EQ-5D-5 L questionnaire had a high reliability with high observed internal consistency (Cronbach’s alpha = 0.816, CI: 0.719–0.886). It showed strong temporal stability, with ICCs of the EQ-5D-5 L score, index and EQ-visual analog scale (EQ-VAS) of 0.852, 0.801, and 0.839 respectively. Agreement by kappa was moderate; above 0.4, for all domains, except for the “Usual activities” domain. EQ-5D-5 L domains, VAS and index had moderate to strong significant correlations with SF-36 and ODI subscales and total scores in the correct direction indicating a good criterion validity of the instrument.

**Conclusion:**

The Arabic version of EQ-5D-5 L is reliable and valid for assessment of HRQoL of Arabic speaking patients.

## Background

Health-related quality of life (HRQoL) measures an individual´s subjective opinion regarding his or her own health in physical, psychological and social dimensions. The measurement of HRQoL has been of great concern for decades, but now, there is a renewed vigour at making quality the organising principle for estimating the outcomes of health care programs and interventions [1, 2]. With increasing concerns about centered patients’ outcomes and prioritizing patients’ subjective perception of well-being after completing the treatment, HRQoL instruments must be added to traditional measurements for comprehensive assessment, taking into consideration the general health of patients, their ability to work, their expectations, and their perception of pain [2, 3].

HRQoL assessment also has an important role in broadening the decisions made by the health team, as well as in comparing quality of life in different cultures and the efficiency of different treatment techniques [4]. It also helps to analyze the effectiveness of clinical interventions and plan health services [5].

Degenerative spine diseases, associated with a variety of clinical symptoms, including lower extremity pain, weakness, and low back pain, cause functional limitations and difficulties in daily living activities of affected patients, with negative impacts on patients’ HRQoL [6, 7].

The European quality of life five dimensions– five levels (EQ-5D-5 L) questionnaire is a commonly used generic questionnaires for assessing HRQoL. It assesses important aspects of health in patients across five dimensions which are; mobility, self-care, usual activities, pain/discomfort and anxiety/depression, which are important aspects influencing patients’ activities and emotional status, hence, their perception about their health and well-being. It is designed for self-completion by respondents and is ideally suited for use in postal surveys, in clinics, and face-to-face interviews. It is cognitively undemanding, taking only a few minutes to complete. It has been developed to generate an index value for HRQoL, reflecting the subjective valuation of health states based on the respondents’ preferences. In addition, the availability of its web-based administration by multiple devices, such as personal computer, tablet, or smartphones, makes this instrument adequate for monitoring the HRQoL in e-Health programs [8].

Based on the findings of recent systematic reviews and an international panel of experts, the EQ-5D-5 L was recommended for use in patients with low back pain [9, 10]. There are more than 170 different language versions of the EQ-5D-5 L produced using a standardized translation protocol [11]. However, despite being validated into many languages [12], a validated Arabic version is missing, yet needed to measure the effect of clinical interventions and to assess the outcomes of patients’ health care on Arab patients’ quality of life, especially that Arabic is considered the 5th most spoken language worldwide [13]. Nevertheless, this will allow comparing study results produced in Arab countries to those produced in different countries in systematic reviews and meta-analysis.

The aim of this study was to test the reliability and validity of the Arabic version of EQ-5D-5 L, so that it would be used as a quality-of-life assessment tool for Arabic-speaking patients.

## Methods

### Study participants and setting

A validation study of the self-reported outcome measure was done on 100 patients. Inclusion criteria were; patients aged 18–60 years old who underwent operative intervention for degenerative spine diseases with a postoperative duration ranging between two to three weeks. Exclusion criteria were as follows; immediate post-operative patients, conservatively treated patients and illiterate patients or patients with problems that interfere with adequate understanding of the questionnaire.

### Recruitment of the study participants

Study participants were recruited from the outpatient spine clinic of Assiut University Hospital. They were invited to participate in the study during their postoperative follow up visits, after clarifying the study objectives. Fifty patients were asked to come for a second visit for the test- retest of the tool. They were contacted by phone calls and text messages for reminding them about the timing of the second visit, and to schedule another visit if they missed the scheduled appointment. The interval of one week was chosen to maintain health status between the two occasions.

### Sample size

The sample size was calculated using Power analysis. For assessment of the reliability, a sample size of 50 allows a 95% CI for an intraclass correlation coefficient of 0.7 to be estimated. For testing validity, a sample size of 92 was calculated, considering a correlation coefficient of 0.7, an alpha error of 0.05 and a power of 90%. The sample size was increased to 100 participants.

### Stages of the study

#### Obtaining the translated arabic version

An Arabic version of the EQ-5D-5 L questionnaire was requested from the Euroqol office website. They provided a version translated into “classic Arabic”, justifying that it is a simple translation that can be easily understood by all levels of education, with no need for providing a “colloquial Arabic” version.

#### Cognitive testing

Cognitive testing of the provided translated version was done on 10 patients by asking them to fill in the questionnaire and mention any difficulty experienced in answering the translated questions and to circle the words or sentences which were ambiguous, confusing or difficult to understand.

#### Testing reliability and validity

The self-administered translated tool was completed by 100 patients. 50 patients were consented to complete the questionnaire in a second follow up visit, at least one week apart, for assessment of the test-retest reliability over time.

The criterion validity was assessed by comparing the responses of 100 patients to the EQ-5D-5 L with comparable subscale scores of two questionnaires obtained at the same time; the validated Arabic version of the Oswestry Disability Index (ODI), which is considered the gold standard of low back functional outcome tools [14], and the validated Arabic version of SF-36 [15], as a generic instrument which has been shown to cover similar areas of HRQoL.

The construct validity was assessed using known-groups comparisons to test for hypothesized differences concerning demographic and clinical variables.

#### Data collection instruments

Demographic data of the participants were collected, including age, sex, residence, educational level and working status. Their diagnoses and performed spine operations were also recorded. They were also asked to complete a structured questionnaire which consisted of the translated Arabic version of EQ-5D-5 L and the validated Arabic versions of SF-36 and Oswestry disability index (ODI).

##### EQ-5D-5 L

The EQ-5D-5L is a brief, multi-attribute, generic, health status measure composed of 5 questions with Likert response options (descriptive system) and a visual analog scale (EQ-VAS). The descriptive system covers 5 dimensions of health; self-care, mobility, usual tasks, pain/discomfort, and anxiety/depression. Each dimension has five levels of severity; no problems, slight problems, moderate problems, severe problems, and extreme problems. The visual analogue scale (EQ-VAS) asks the participants to rate their health on the day from 0 (‘the worst health state you can imagine’) to 100 (the best health state you can imagine’). The scores of the five domains are combined into a five-digit number which is converted into a single index value [8].

##### ODI

The ODI is comprised of ten items with associated statements for the patient to select which reflect the patient’s ability to manage their everyday life while dealing with their pain. Each of the ten items in the ODI has six response options from which patients are requested to select one which indicates his/her health status. This allows scoring from 0 to 5 for each item, with lower scores indicating less pain and disability [14].

##### SF-36

SF-36 is a generic instrument that assesses a patient’s HRQoL along with eight subscales and two summary scales. It consists of 36 items measuring the following eight domains: four domains in the area of physical health; physical functioning (PF), role physical (RP), general health (GH), and bodily pain (BP) and four domains in the area of mental health; role emotional (RE), vitality (VT), mental health (MH), social functioning (SF). The summary scales consist of a physical component (PCS) and a mental component (MCS). The SF-36 subscales and summary scores range from 0 to 100, with higher scores reflecting better HRQoL [15, 16].

#### Statistical analysis

Data entry was done using MS Excel 2013. Statistical analysis was done using the IBM SPSS 23.0 (IBM SPSS Inc., Chicago, IL, USA). Descriptive statistics were performed using mean and standard deviations for quantitative scores, frequencies, and percentages for the qualitative domains of the instrument. EQ-5D-5 L total score was calculated by summation of the score values of the five domains of the questionnaire. For calculating the EQ-5D-5 L index values, in the absence of a country-specific crosswalk/value set, we used the most frequently used value set (UK), which was available on the EuroQol website based on the recommendation of the EuroQol office. Single preference-based indices were produced ranging from 1 (the best health state) to negative values (health states valued as worse than death), where 0 = death.

Test-retest reliability was determined using the intra-class correlation coefficient (ICC) for the EQ-5D-5 L score, EQ-VAS, and EQ-5D-5 L index, using the two-way mixed method. An ICC above 0.7 was considered acceptable [17]. The degree of agreement for the EQ-5D-5 L self-classifier domains was evaluated by Cohen’s kappa statistic. A weighted Kappa score of < 0.2 was indicative of poor agreement, 0.21–0.4 was fair, 0.41–0.6 was moderate, 0.61–0.8 was good, and ≥ 0.8 was very good [18]. Percent of agreement was also calculated. The difference between the EQ-5D-5 L score at baseline and one week later was calculated, a one-sample t-test was performed to compare the difference between the two ratings with a “Zero” value, which indicates “No difference”. Mean difference and SD were used to calculate the upper and lower bounds of 95% CI of the mean difference and draw a Bland-Altman Plot. The internal consistency was assessed by Cronbach’s alpha [19].

To assess the criterion validity, we analyzed whether the response levels of the EQ-5D-5 L self-classifier were associated with different comparable SF-36 and ODI scores. For the EQ-VAS score and EQ-5D-5 L index, Spearman correlation coefficients with the SF- 36 and ODI subscales were calculated. The construct validity was assessed using known-groups comparison to test for the differences in the hypothetical variables in relation to EQ-5D-5 L total score, VAS and index. Statistical significance was defined as a p-value less than 0.05 for all statistical tests.

## Results

### Acceptability and cognitive assessment

Acceptance of the EQ-5D-5 L self-classifier and EQ-VAS were assessed by the proportion of missing or invalid responses. Invalid responses are those where the participants select more than one response or make an ambiguous mark. In the total sample, the proportion of missing or invalid responses to the EQ-5D-5 L self-classifier ranged from 1 to 2% for the single items and was 2% for the EQ-VAS. The questionnaire filling time ranged between 4 and 8 min. None of the patients reported the inability to complete the questionnaire because of linguistic problems.

### Characteristics of the patients

The mean age of the study participants was 41.27 ± 9.17 years ( range between 22 and 55 years). 58% were males while 42% were females. 25% of patients had below secondary education. Most patients were working (68%). More than half of the participants had lumbar disc prolapse (60%) and about half of participants underwent discectomy (52%) (Table 1).

**Table 1:** Demographic characteristics of the study patients

Variables	*N* = 100
**Gender**	
Male	58 (58%)
Female	42 (42%)
**Age (years)**	
(Mean ± SD)	41.27 ± 9.17
Range	(22–55)
**Residence**	
Rural	34 (34%)
Urban	66 (66%)
Education level	
Read and write	10 (10%)
Basic education	15 (15%)
Secondary education	35 (35%)
Above secondary education	40 (40%)
**Working status**	
Working	68 (68%)
Not working	32 (32%)
**Diagnosis**	
Lumbar disc prolapse (LDP)	60 (60%)
Lumbar canal stenosis (LCS)	10 (10%)
Spondylolisthesis	22 (22%)
Spondylolysis	8 (8%)
**Type of surgery**	
Discectomy	52 (52%)
Fusion	31 (31%)
Decompression	9 (9%)
Discectomy with fusion	7 (7%)
Modified Scott	1 (1%)

### Reliability of the arabic version of EQ-5D-5 L

The Arabic version of EQ-5D-5 L questionnaire had a high reliability with high observed internal consistency, as Cronbach’s alpha of the questionnaire was (0.816) with 95% CI (0.719–0.886).

The Arabic version of EQ-5D-5 L showed strong temporal stability as the ICCs of the EQ-5D-5 L score, index and EQ-VAS were 0.852, 0.801, and 0.839 respectively. For the EQ-5D-5 L self-classifier, agreement by kappa was moderate; above 0.4, for all domains, except for the “Usual activities” domain (0.35). The percent of agreement between the two ratings ranged from 42.9% for “usual activities” to 65.3% for “Anxiety/ Depression”. The item total correlation coefficients show that mobility, self-care, usual activities, and pain scores are moderately correlated with the average score of the remaining domains (*r* = 0.652, 0.719, 0.712, and 0.651 respectively) while only the “depression” domain showed a weak correlation (*r* = 0.387) (Table 2).

**Table 2:** Test-retest reliability of domains and total scores of the EQ-5D-5 L

	Weighted Kappa	Percent of agreement (%)	Corrected Item-total correlation
**EQ-5D-5 L domain**			
• Mobility	0.473	51.0	0.652
• Self-care	0.434	63.3	0.719
• Usual activities	0.346	42.9	0.712
• Pain	0.471	51.0	0.651
• Depression	0.538	65.3	0.387
**ICC (95% CI)**			
EQ-5D-5 L score	0.852 (0.752–0.914)		
EQ_VAS	0.839 (0.732–0.906)		
EQ-index	0.801 (0.673–0.883)		

**Fig. 1:**
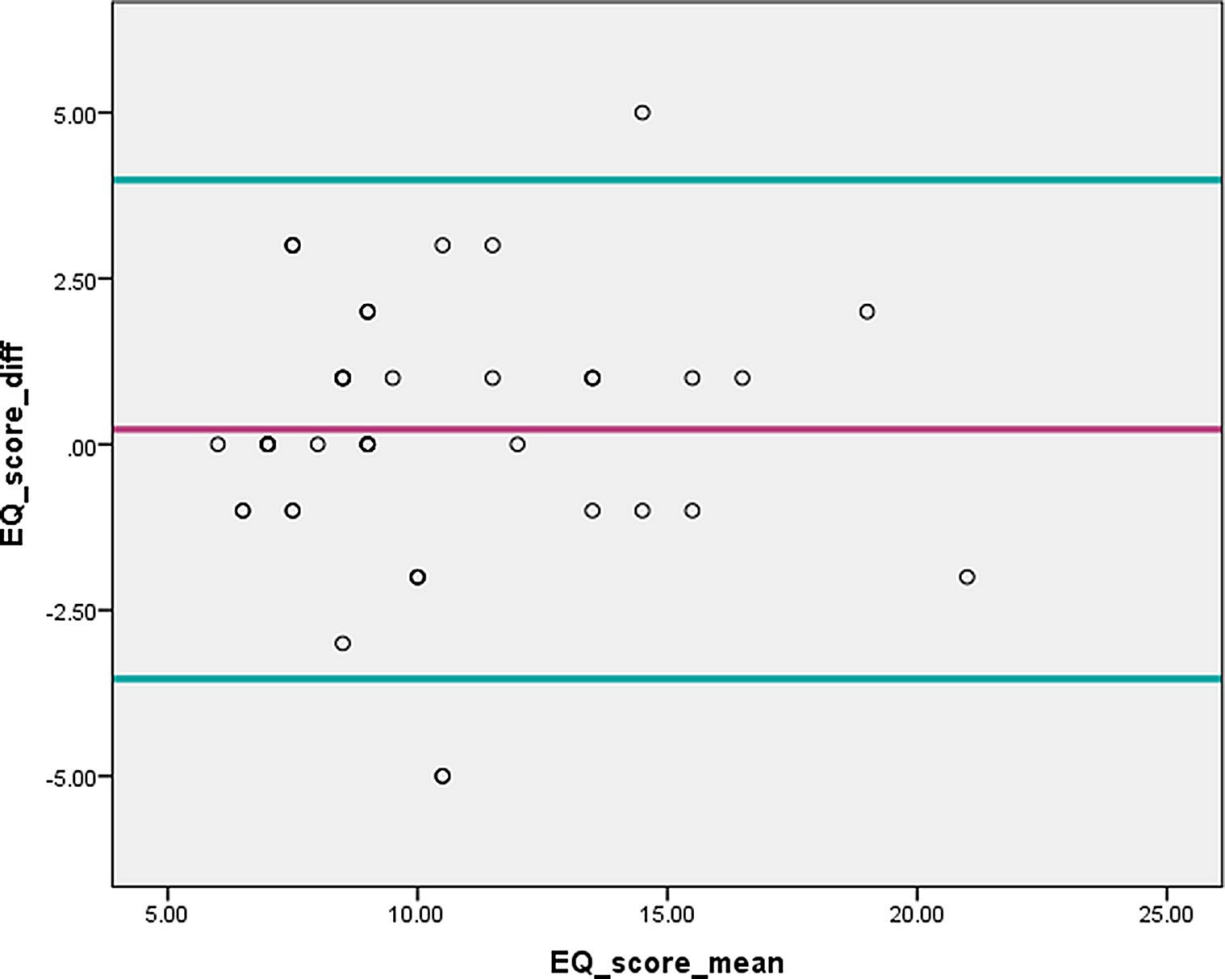
Bland Altman Plot of the agreement between the EQ-5D-5 L score at baseline and one week later

The horizontal lines of Bland Altman plot were drawn at the mean difference (0. 224), and at the limits of agreement between the baseline rating and the second rating. The graph shows that limits of agreement did not exceed the maximum allowed difference between ratings; 95% of the data points lie within ± 2 SD of the mean difference, which indicates that the two ratings may be used interchangeably and hence indicates the tool reliability (Fig. 1).

### Validity of the arabic version of EQ-5D-5 L

**Fig. 2:**
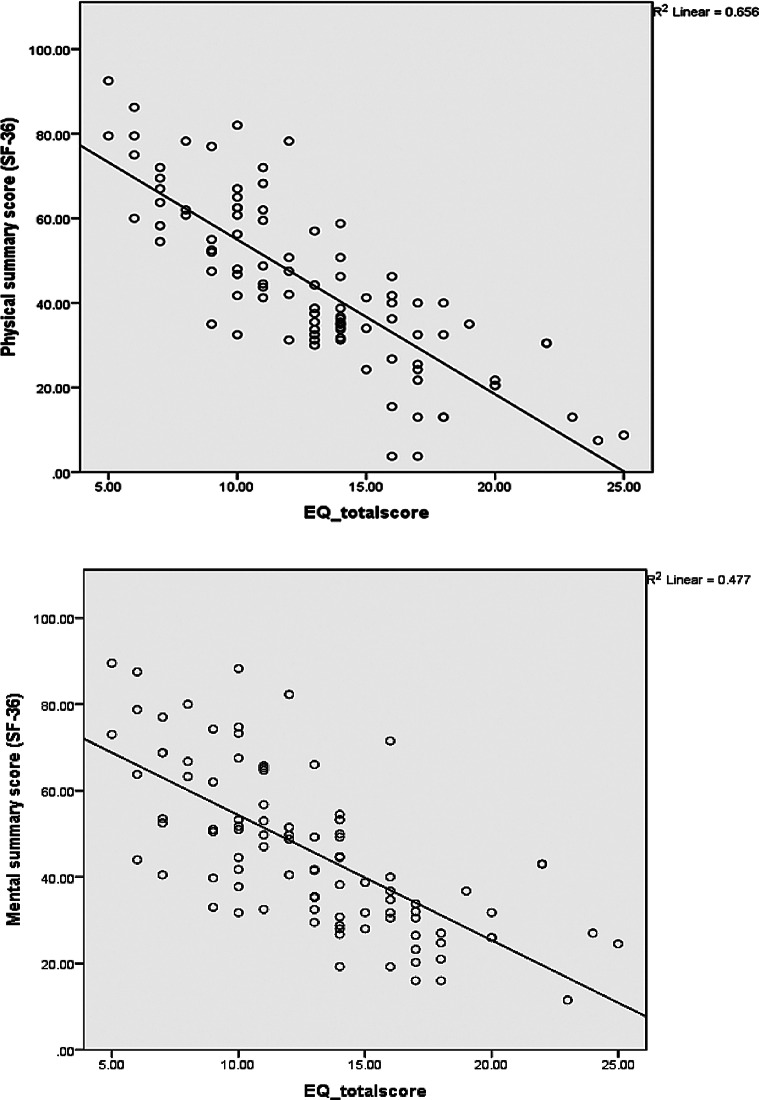
Scatter Plot diagram of the correlation between physical and mental summary scores of SF-36 and EQ-5D-5 L-total score

**Fig. 3:**
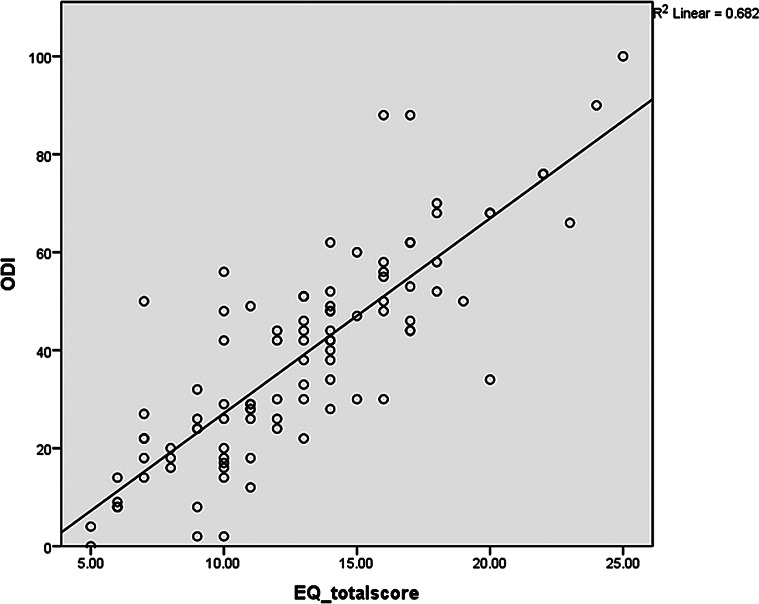
A scatter plot of the correlation between EQ-5D-5 L total score and ODI score

The criterion validity for EQ-5D-5 L was assessed by comparing participants, classified by EQ-5D-5 L, as having no/slight problems and those having moderate/extreme problems with their SF-36 and ODI scores. Tables 3 and 4 shows that the SF-36 dimensions scores were significantly higher; indicating better HRQoL, among participants with no/slight problems as compared to participants with moderate to extreme problems, while the ODI total score and dimensions scores were higher among participants with moderate to extreme problems, as compared to participants with no/slight problems, as a high ODI score indicates a poorer quality of life in line with the EQ-5D-5 L questionnaire scores. Figure 2 also illustrates the negative strong correlation (*r* = − 0.809) and the negative moderate correlation (*r* = − 0.691) between EQ-5D-5 L total score and the physical and mental summary scores of SF-36, respectively. Figure 3 also shows a strong positive correlation between ODI and EQ-5D-5 L total scores (*r* = 0.825).

**Table 3:** Correlation between EQ-5D-5 L and SF-36 domains scores

	Physical functioning	Physical health	Emotional health	Energy/fatigue	Emotional well being	Social functioning	Pain	General health
**Mobility**								
No/slight problem	61.34 ± 22.30**	52.44 ± 35.70**	54.46 ± 40.70*	52.76 ± 16.94*	60.20 ± 16.87*	69.27 ± 14.57**	66.20 ± 18.14**	61.95 ± 13.27**
Moderate/extreme	36.16 ± 22.07	17.98 ± 23.05	24.56 ± 30.63	35.09 ± 13.41	43.23 ± 14.86	40.84 ± 15.27	34.84 ± 15.83	40.96 ± 14.46
**Self-care**								
No/slight problem	51.27 ± 24.99**	43.65 ± 34.77**	47.62 ± 38.72**	46.56 ± 17.87**	54.52 ± 18.02**	59.62 ± 19.56**	56.40 ± 21.41**	56.83 ± 14.03**
Moderate/extreme	36.56 ± 24.39	11.81 ± 19.35	19.44 ± 29.20	34.44 ± 14.02	42.36 ± 15.18	38.56 ± 17.04	31.31 ± 16.71	37.22 ± 15.83
**Usual activities**								
No/slight problem	62.08 ± 24.15**	51.39 ± 21.03**	54.67 ± 40.80*	51.39 ± 18.22**	58.44 ± 18.98**	68.36 ± 15.73**	65.53 ± 20.12**	60.65 ± 14.13**
Moderate/extreme	37.32 ± 21.69	21.03 ± 26.64	26.43 ± 32.37	37.11 ± 14.48	45.56 ± 15.17	42.97 ± 18.02	37.16 ± 18.10	43.65 ± 15.94
**Pain**								
No/slight problem	69.64 ± 20.18**	51.79 ± 41.90**	51.21 ± 43.99**	53.39 ± 20.14**	59.86 ± 20.58**	70.69 ± 14.53**	68.71 ± 19.08**	59.82 ± 15.48**
Moderate/extreme	36.82 ± 21.19	23.96 ± 26.35	31.47 ± 34.04	37.54 ± 14.20	46.11 ± 15.26	44.53 ± 18.58	39.18 ± 18.96	45.49 ± 16.59
**Depression**								
No/slight problem	53.07 ± 24.72*	44.07 ± 33.90*	49.73 ± 37.90**	47.25 ± 17.97*	57.68 ± 17.01**	59.19 ± 19.39**	56.85 ± 21.15**	58.14 ± 14.16**
Moderate/extreme	35.85 ± 23.44	14.02 ± 23.4.39	18.68 ± 29.89	34.39 ± 13.79	38.85 ± 12.69	41.49 ± 19.31	33.93 ± 18.89	37.07 ± 13.96

*P < 0.01

**P < 0.001

**Table 4:** Correlation between EQ-5D-5 L and ODI total and domains scores

	ODI Total score	Pain	Self-care	Lifting	Walking	Sitting	Standing	Sleeping	Sex	Social life	Travelling
**Mobility**											
No/slight problem	29.91 ± 16.02**	1.59 ± 0.91**	1.0 ± 0.91**	2.05 ± 1.39	1.46 ± 0.91**	1.24 ± 0.86*	1.69 ± 1.08*	1.17 ± 0.95**	1.05 ± 0.88**	1.44 ± 1.08**	1.62 ± 1.30**
Moderate/extreme	52.34 ± 20.45	2.85 ± 1.1	2.21 ± 1.32	2.95 ± 1.49	2.71 ± 1.28	2.46 ± 1.20	2.66 ± 1.15	2.17 ± 1.160	2.76 ± 1.41	2.77 ± 0.95	3.10 ± 1.46
**Self-care**											
No/slight problem	30.13 ± 16.26**	1.62 ± 0.91**	0.95 ± 0.85**	2.03 ± 1.43	1.44 ± 0.91*	1.27 ± 0.87*	1.71 ± 1.08**	1.19 ± 0.99**	1.24 ± 1.20*	1.48 ± 1.07**	1.58 ± 1.16**
Moderate/ extreme	54.72 ± 19.67	2.94 ± 1.14	2.39 ± 1.22	3.06 ± 1.3	2.89 ± 1.23	2.56 ± 1.22	2.75 ± 1.15	2.28 ± 1.08	2.72 ± 1.30	2.88 ± 0.94	3.40 ± 1.47
**Usual activities**											
No/slight problem	22.33 ± 14.30*	1.06 ± 0.63**	0.61 ± 0.8**	1.53 ± 1.32	1.06 ± 0.83*	0.97 ± 0.84*	1.28 ± 1.05*	0.81 ± 0.82**	0.79 ± 0.55*	1.08 ± 0.94**	1.14 ± 1.0**
Moderate / extreme	48.65 ± 18.29	2.71 ± 0.99	1.97 ± 1.16	2.90 ± 1.36	2.49 ± 1.14	2.17 ± 1.12	2.54 ± 1.04	2.00 ± 1.07	2.61 ± 1.39	2.48 ± 1.07	2.84 ± 1.48
**Pain**											
No/slight problem	21.04 ± 14.23**	1 ± 0.72**	0.52 ± 0.84**	1.39 ± 1.16	0.96 ± 0.74**	0.89 ± 0.78**	1.18 ± 1.02*	0.86 ± 0.84**	0.70 ± 0.55**	0.93 ± 0.85**	0.85 ± 0.60**
Moderate / extreme	46.14 ± 18.99	2.54 ± 1.03	1.85 ± 1.1	2.82 ± 1.42	2.36 ± 1.17	2.07 ± 1.14	2.44 ± 1.08	0.70 ± 0.55	2.43 ± 1.39	2.75 ± 1.84	2.75 ± 1.48
**Depression**											
No/slight problem	21.93 ± 12.94**	1.20 ± 0.64	0.68 ± 0.85*	1.44 ± 1.30	1.12 ± 0.93**	0.93 ± 0.75**	1.20 ± 0.93*	0.90 ± 1.02**	0.75 ± 0.56*	1.12 ± 0.95**	1.05 ± 0.87**
Moderate / extreme	50.96 ± 17.05	2.72 ± 1.01	2.04 ± 1.1	3.11 ± 1.2	2.60 ± 1.08	2.28 ± 1.08	2.68 ± 0.96	1.98 ± 0.91	2.79 ± 1.27	2.57 ± 0.95	3.05 ± 1.39

*P < 0.01

**P < 0.001

Table 5 shows that the EQ-VAS had a positive significant spearman correlation with all parameters of the SF-36 questionnaire, which were considered moderate to strong (r ranged between 0.4 and 0.69, *p* < 0.001), except for emotional well-being (*r* = 0.37, *p* < 0.001). There was also a negative strong correlation between the EQ-VAS scores and ODI total score (*r* = -0.73, *p* < 0.001). Regarding the EQ-5D-5 L index, it had a moderate to strong negative significant correlation with all parameters of the SF-36 questionnaire (r ranging between − 0.45 to -0.68, *p* < 0.001), and weak positive correlation with ODI total score (0.36, *p* < 0.001).

**Table 5:** Correlation of SF-36 and ODI scores with EQ-VAS and EQ-index

	EQ-VAS	EQ index
	r value	r value
Physical functioning	0.40*	-0.49*
Limitation due to physical healthy	0.45*	-0.50*
Limitation due to emotional heath	0.46*	-0.34*
Energy/fatigue	0.41*	-0.51*
Emotional well-being	0.37*	-0.45*
Social functioning	0.58*	-0.63*
Pain	0.65*	-0.65*
General health	0.69*	-0.65*
Physical summary score	0.57*	-0.68*
Mental summary score	0.66*	-0.58*
ODI score	-0.73*	0.36*

*P < 0.001

Known group comparisons showed that none of the participants’ demographic characteristics was associated significantly with different EQ-5D-5 L measures. Only the postoperative duration showed significant moderate negative correlation with the EQ-5D-5 L score (*r* = − 0.41, *p* < 0.001). Postoperative duration showed also a moderate positive correlation with VAS (*r* = 0.45, *p* < 0.001).

## Discussion

The study results indicate that the translated Arabic version of EQ-5D-5 L has good psychometric property, and can be used as an assessment tool for quality of life in the Arab speaking population. It has the advantage of being a generic instrument, as it captures a very broad range of health statuses, permit comparisons between patient groups and can be broadly applicable across specialties and populations [20]. For spine patients, all domains of EQ-5D-5 L; mobility, self-care, usual activities, pain and depression are important and could represent functional limitation, affecting their perception of well-being.

As regards cognitive assessment of the questionnaire, questions were found to be easily understood and accepted by participants of all educational levels, as none of the study participants reported the inability to complete the questionnaire. This was supported by the low proportion of missing and invalid entries for the EQ-5D-5 L domains as well as for the EQ-VAS. Similar reactions were observed in comprehension and fulfillment of the Chinese version of the EQ-5D-5 L questionnaire [21].

The Arabic version demonstrated excellent internal consistency with a cronbach’s alpha of 0.816. This indicates good internal agreement between the tool items and does not suggest that any of the items was redundant. Stable test-retest reliability was also observed, as the ICC of the EQ-5D-5 L score, index, and EQ-VAS were 0.852, 0.801, and 0.839 respectively. The inter-item correlation coefficient for the self-classifier domains showed that the total EQ-5D-5 L score was moderately correlated with mobility, self-care, usual activities, and pain domains (*r* = 0.652, 0.719, 0.712, and 0.651 respectively) and weakly correlated with depression (*r* = 0.387), which indicates that all items in the scale are measuring the same constructs and that the instrument is reliable. The reliability of the instrument was also supported by the Kappa statistic for the qualitative categories of EQ-5D-5 L self-classifier. These results agree with those found in previous studies using translated tools in other languages; Spanish and Chinese versions, and used for other diseases, such as adolescent idiopathic scoliosis, osteoarthritis [21–24].

The different domains of the Arabic version of the EQ-5D-5 L were strongly correlated with ODI and the SF-36 components, which are validated tools commonly used to assess quality of life, indicating a good criterion validity. The SF-36 was used as a criterion as it has been successfully tested and repeatedly used as a generic measure assessing HRQoL and it covers similar areas assessed by EQ-5D-5 L. ODI is also considered the gold standard of low back functional outcome tools.

Moderate to strong positive correlations were observed between the EQ-VAS score and the health domain of the SF-36, except for emotional well-being. This can be attributed to the expected fluctuation in emotional conditions affected by any other incident event, other than the health condition. The positive observed correlation indicates a right direction of correlation as higher VAS scores indicate better quality of life, in line with higher SF-36 scores. There was also a negative strong correlation between the EQ-VAS scores and ODI total score. This opposite direction supports the agreement between the ratings of the two instruments, as higher EQ-VAS indicate better quality of life, while higher ODI scores indicate poorer quality of life.

Our results were consistent with previous studies, as a validation study of the German version of EQ-5D-5 L among cardiac rehabilitation patients and another study using the German version on patients with inflammatory bowel diseases showed excellent criterion validity, when comparing EQ-index and VAS scores with the comparable SF-36 domain scores [25, 26]. Another study which assessed the psychometric properties of EQ- 5D-5 L in low back pain patients showed also strong correlations between EQ-5D-5 L and SF -6D scores [27]. The strong correlation with ODI scores was also detected in other studies [23, 28].

Known group comparisons showed that none of the participants’ demographic characteristics was associated significantly with different EQ-5D-5 L measures. However, the postoperative duration showed significant moderate negative correlation with the EQ-5D-5 L score (*r* = − 0.41, *p* < 0.001). This can be explained by less perception of problems in different dimensions of the tool with increasing the postoperative duration that could reflect an improvement in the patients’ perception of health status. Postoperative duration showed also a moderate positive correlation with VAS (*r* = 0.45, *p* < 0.001), as the increase of postoperative duration is associated with a concomitant increase of VAS, and hence better HRQoL perception.

### Strengths and limitations

The EQ_5D-5 L is one of the most commonly used generic preference-based HRQoL measures and is validated in many languages. Our study has provided a validated translated Arabic version, which has been previously unavailable. Comparing EQ-5D-5 L with a generic instrument; SF-36 and a specific instrument for low back pain; ODI, strengthened the study. However, there were some limitations. We used a convenience sample of a relatively small size, which may restrain the generalizability of our findings. Further studies are required to evaluate the reliability and validity of other forms of EQ-5D-5 L such as the self-complete form and the web-based form and to test the tool in other Arabic-speaking countries.

## Conclusion

The Arabic EQ-5D-5 L was shown to be valid and reliable in evaluating HRQoL in Egypt and thus can be used as a tool for patients whose primary language is Arabic.

## Data Availability

The datasets used and/or analysed during the current study is available from the corresponding author on reasonable request.
